# The rate of the molecular clock and the cost of gratuitous protein synthesis

**DOI:** 10.1186/gb-2010-11-9-r98

**Published:** 2010-09-29

**Authors:** Germán Plata, Max E Gottesman, Dennis Vitkup

**Affiliations:** 1 Center for Computational Biology and Bioinformatics, Columbia University, 1130 St Nicholas Ave, New York City, NY 10032, USA; 2 Integrated Program in Cellular, Molecular, Structural, and Genetic Studies, Columbia University, 1130 St Nicholas Ave, New York City, NY 10032, USA; 3 Department of Microbiology and Immunology, Columbia University, 701 W. 168 St, New York City, NY 10032, USA; 4 Department of Biochemistry and Molecular Biophysics, Columbia University, 701 W. 168 St, New York City, NY 10032, USA; 5 Department of Biomedical Informatics, Columbia University, 1130 St Nicholas Ave, New York City, NY 10032, USA

## Abstract

**Background:**

The nature of the protein molecular clock, the protein-specific rate of amino acid substitutions, is among the central questions of molecular evolution. Protein expression level is the dominant determinant of the clock rate in a number of organisms. It has been suggested that highly expressed proteins evolve slowly in all species mainly to maintain robustness to translation errors that generate toxic misfolded proteins. Here we investigate this hypothesis experimentally by comparing the growth rate of *Escherichia coli *expressing wild type and misfolding-prone variants of the LacZ protein.

**Results:**

We show that the cost of toxic protein misfolding is small compared to other costs associated with protein synthesis. Complementary computational analyses demonstrate that there is also a relatively weaker, but statistically significant, selection for increasing solubility and polarity in highly expressed *E. coli *proteins.

**Conclusions:**

Although we cannot rule out the possibility that selection against misfolding toxicity significantly affects the protein clock in species other than *E. coli*, our results suggest that it is unlikely to be the dominant and universal factor determining the clock rate in all organisms. We find that in this bacterium other costs associated with protein synthesis are likely to play an important role. Interestingly, our experiments also suggest significant costs associated with volume effects, such as jamming of the cellular environment with unnecessary proteins.

## Background

Once the first protein sequences became available, their comparison led to the conclusion that the number of accumulated substitutions between orthologs was mainly a function of the evolutionary time elapsed since the last common ancestor of corresponding species [1,2]. Consequently, orthologous proteins accumulate substitutions at an approximately constant rate over long evolutionary intervals. This observation suggests that one can use available protein sequences as a molecular clock to estimate divergence times between different species [3]. Further studies revealed that while the pace of the molecular clock is similar for orthologous proteins in different lineages, it varies by several orders of magnitude across non-orthologous proteins [4,5].

For several decades the dominant hypothesis explaining the large variability of the molecular clock rate between non-orthologous proteins was based on the concept of functional protein density: the higher the fraction of protein residues directly involved in its function, the slower the protein molecular clock [6,7]. It was not until high-throughput genomics data became widely available that multiple molecular and genetic variables were used to investigate the dominant factors influencing the molecular clock rates of different proteins. Surprisingly, such features as gene essentiality [8-11], the number of protein-protein interactions [12,13], and specific functional roles [14,15], have been shown to have, on average, either non-significant or significant but relatively weak correlations with protein evolutionary rates. On the other hand, quantities directly related to gene expression, such as codon bias, mRNA expression, and protein abundance, showed the strongest correlation with the rate of protein evolution [16,17]. For example, expression alone explains about a third of the variance in the substitution rates in several microbial species [14,17,18] and about a quarter of the variance in *Caenorhabditis elegans *[19]. In these and many other organisms, highly expressed genes accept significantly less synonymous and non-synonymous (amino acid changing) substitutions than genes with low expression levels [20].

Considering the major role played by expression in setting the rate of amino acid substitutions, it is important to understand the main molecular mechanisms of this effect [21]. A popular theory by Drummond *et al. *[18,22,23] suggests that highly expressed proteins may evolve slowly in all organisms, from microbes to human [22], due to the selection against toxicity associated with protein misfolding. The logic behind this interesting hypothesis is that a significant fraction (> 10%) of cellular proteins may contain translation errors [24,25] that could cause cytotoxic protein misfolding. If misfolded proteins indeed incur substantial toxicity costs, greater pressure to avoid misfolding will affect highly expressed genes since they generate relatively more misfolded proteins [18]. Consequently, adaptive pressure will maintain sequences of highly expressed proteins robust to translation errors, which will in turn slow the amino acid substitution rate, that is, the protein molecular clock. The misfolding toxicity hypothesis was supported by the results of computer simulations [22], but to the best of our knowledge, it has never been tested experimentally.

In this study we specifically investigated whether the toxicity of misfolded proteins or other costs associated with protein synthesis make a dominant contribution to cellular fitness (growth rate), and consequently constrain the molecular clock in *Escherichia coli*. To test this, we used wild type (WT) and misfolding-prone variants of the *E. coli *β-galactosidase gene, *lacZ*. We also computationally analyzed the contribution of other related factors, such as protein stability and solubility.

## Results

The native biological function of the LacZ protein is to cleave lactose for use as a source of carbon and energy [26]; in the absence of lactose, β-galactosidase does not participate in *E. coli *carbon metabolism. Therefore, we used *lacZ *expression in a lactose-free medium to measure the cost of gratuitous protein expression [27,28]. To compare that expression cost to the cost of potentially toxic protein misfolding, we used site-directed mutagenesis to engineer several destabilizing single-residue substitutions into LacZ. Single amino acid substitutions should serve as a good model for translational errors because only rarely, in about 10% of the proteins that contain translation errors, two or more residues will be simultaneously mistranslated in the same protein. We expressed the misfolding-prone mutants at the same level as the WT protein. Because the misfolded LacZ proteins are both potentially toxic and also devoid of biological function, the comparison of the growth rates of bacteria carrying the WT and each of the destabilized mutants allowed us to evaluate the additional fitness cost specifically arising from misfolding toxicity.

### Destabilizing mutations in *lacZ *yield aggregated and partialy soluble proteins

Amino acid substitutions in protein cores are significantly more destabilizing than substitutions on protein surfaces [29,30]. Therefore, we selected five buried residues encoding non-polar amino acids that could be mutated to polar residues with single nucleotide substitutions while maintaining a similar level of codon preference (Table 1). We used the DPX server [31] to identify buried residues of the LacZ protein based on its crystal structure (Protein Data Bank (PDB) code 1dp0). We then applied the I-Mutant2.0 algorithm [32] to confirm that the selected substitutions would be indeed destabilizing. Using site-directed mutagenesis, the five selected substitutions were introduced separately into plasmids containing *lacZ *under transcriptional control of the isopropyl β-D-1-thiogalactopyranoside (IPTG)-inducible *lac *promoter [33]. We then used a β-galactosidase assay [34] to experimentally confirm reductions in the catalytic activity of LacZ in all of the generated mutants (Table 1).

**Table 1:** Characteristics of destabilizing mutations engineered into *E. coli *β-galactosidase

	Mutant				
	
	V567D	F758S	I141N	G353D	A880E
Predicted ΔΔG (kcal/mol)	-2.6	-2.9	-2.4	-1.6	-0.6
Relative protein activity (%)	31	4	17	2	61
Codon substitution (WT/mutant)	GTC/GAC	TTT/TCT	ATT/AAT	GGC/GAC	GCG/GAG
Codon preference % (WT/mutant)	13.5/53.9	29.0/32.4	33.5/17.3	42.8/53.9	32.3/24.7
Found in inclusion bodies (see Figure 1a)	No	Yes	Yes	Yes	No

In the table, ΔΔG values represent destabilizing effects predicted by the I-Mutant2.0 server [32]. The experimentally determined enzymatic activities of the mutants (in percentages) are shown in the table relative to WT.

**Figure 1:**
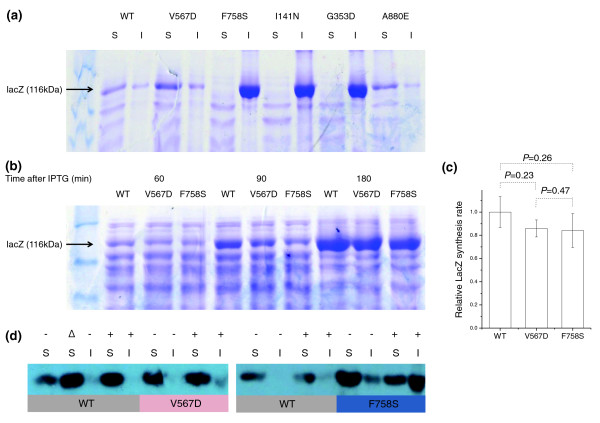
**Expression of destabilizing mutants and wild-type LacZ**. **(a) **SDS-PAGE of soluble and insoluble fractions of cells expressing WT LacZ and five destabilizing mutants induced with 10 μM IPTG. **(b) **Total β-galactosidase at different times after IPTG induction. The LacZ band is indicated by the black arrow. **(c) **Relative synthesis rate of β-galactosidase. *P*-values were obtained using a *t*-test of the linear regression slopes based on quantification of the gel images. Error bars represent the standard error of the regression slopes. **(d) **GroEL western blots in cells exprerssing WT and LacZ mutants. S, soluble fraction; I, insoluble fraction; '-', no IPTG; '+', 20 μM IPTG; Δ, heat shock (1 h shift from 37 to 42°C).

To determine whether the destabilized proteins tended to aggregate, we separated soluble proteins and proteins in inclusion bodies (see Materials and methods) and analyzed them by SDS-PAGE (Figure 1a). The three mutants with the lowest catalytic activity (F758S, I141N and G353D) were found in inclusion bodies (Table 1), the remaining two mutants (V567D and A880E) and WT proteins were found mainly in the soluble protein fraction. Next, by inspecting total cell extracts at different time points after IPTG induction, we confirmed that the total amount of protein synthesized in each mutant strain was similar to that in the WT. As shown in Figure 1b, similar amounts of LacZ are produced in the WT and either soluble (V567D) or insoluble (F758S) mutants. Quantitative analysis of the Coomasie stained bands also did not reveal any significant difference between the LacZ synthesis rates in WT and mutant strains (Figure 1c). Finally, because expression of misfolded proteins is expected to generate a heat shock response [35,36], we used western blots to monitor the amount of the GroEL heat shock protein in induced and un-induced cells carrying WT and mutant *lacZ *(Figure 1d). In cells carrying WT *lacZ*, the concentration of GroEL increased when IPTG was added. However, in both the V567D and F758S mutants, the levels of GroEL in either induced or uninduced cells were equal or higher than that in induced WT cells.

Overall, the results described in this section demonstrate that: all engineered mutants have significantly reduced catalytic activities; soluble and insoluble mutants are expressed at the same level as WT; and the mutants induce a heat shock response, and in some cases aggregate in inclusion bodies.

### Misfolded proteins are no more toxic than wild-type proteins

**Figure 2:**
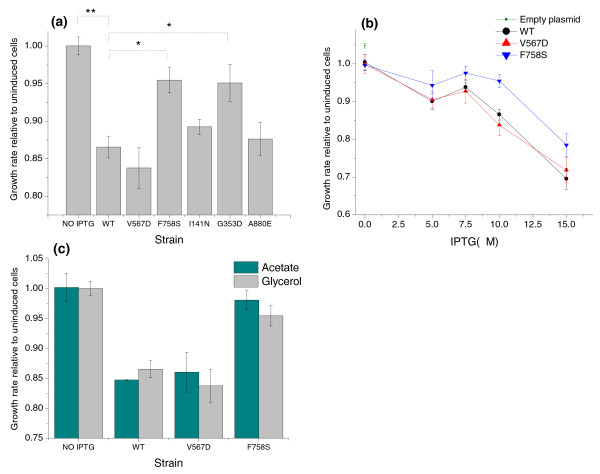
**Comparison of the growth rates for wild-type and misfolding-prone LacZ**. **(a) **Growth rates of cells expressing WT LacZ relative to uninduced cells and cells expressing each of the five destabilizing mutants (10 μM IPTG). Mann-Whitney U *P*-value: *0.02; **8 × 10^-4^. **(b) **Growth rates of cells expressing WT LacZ and two mutants at different induction (IPTG) levels; the growth rate of cells carrying an empty plasmid is also shown for comparison. **(c) **Growth rates of cells expressing LacZ and two destabilizing mutants on acetate and glycerol as the main carbon source; in both cases expression was induced with 10 μM ITPG). Error bars represent the standard error of the mean calculated based on triplicate experiments.

The synthesis of WT or mutant β-galactosidase was initially induced by adding 10 μM IPTG. Using WT LacZ activity as a reference [37], we estimated that about 30,000 molecules of β-galactosidase were present in each bacterial cell at this induction level. This approximately corresponds to half of the protein molecules expressed by a fully induced WT *lacZ *operon [34]. Cells expressing WT LacZ grew 13.5% slower on glycerol as the sole carbon source compared to uninduced cells (Figure 2a). If misfolded proteins indeed impose a significant extra cost on the bacterium, then similarly expressed mutant strains with destabilizing substitutions should lead to a more pronounced growth decrease compared to the one observed with WT LacZ. However, as shown in Figure 2a, the mutant strains grew as well as cells expresing WT LacZ, and, despite inclusion body formation, two of the mutants even grew significantly faster (see Discussion).

To further explore the potential toxicity of the destabilized proteins, we focused on two mutants (F758S and V567D). These mutants are examples of a completely aggregated and a soluble but destabilized LacZ protein, respectively. By varying the concentration of IPTG, we monitored the growth of cells with different levels of expressed LacZ proteins (Figure 2b). Importantly, no additional growth decrease was observed in the mutant strains compared to the WT at all IPTG induction levels. When no IPTG was added, resulting in a low expression level from the un-induced promoter, we also observed the same growth rate reduction in all constructs relative to cells carrying an empty pBR322 plasmid (Figure 2b).

We investigated the possibility that the toxicity of misfolded proteins was more pronounced on a relatively poor carbon source by measuring the growth of the *E. coli *V567D and F758S mutants and the WT on acetate. Although the overall growth rate on acetate was only about 60% of that on glycerol, we again did not observe any additional fitness (growth) decrease due to the destabilizing mutations (Figure 2c). This experiment confirmed that the observed results are not specific to a particular carbon source.

### Nucleotide level selection, protein solubility, and stability in *E. coli*

**Figure 3:**
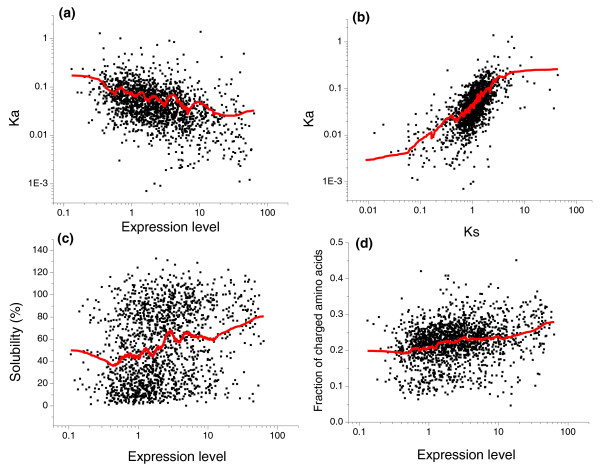
**Correlation of *E. coli *mRNA expression with Ka, protein solubility, and the fraction of charged residues**. **(a) **Correlation between expression and the rate of non-synonymous substitutions (Ka; Spearman's r = -0.45, *P *< 10^-10^). **(b) **Correlation between Ka and the rate of synonymous substitutions (Ks; r = 0.66, *P *< 10^-10^). **(c) **Correlation between expression and protein solubility measured *in vitro *[48] (r = 0.27, *P *< 10^-10^). **(d) **Correlation between expression and the fraction of charged residues (r = 0.28, *P *< 10^-10^). The red lines on each panel represent a 200-point moving average.

Nucleotide sequences of highly expressed genes are significantly constrained by selection for amino acid codons corresponding to abundant tRNAs [38-40]. A recent experimental analysis by Kudla *et al. *[41] suggests that non-optimal codons can directly influence *E. coli *growth (fitness). Using 154 variants of GFP with multiple random synonymous substitutions, these authors found a significant positive correlation between codon optimality and bacterial growth rate. An important role played by the nucleotide-level selection in evolution of *E. coli *proteins is also supported by a high correlation between the rates of non-synonymous (Ka) and synonymous (Ks) substitutions (Figure 3b; Spearman's rank correlation r = 0.66, *P*-value < 10^-10^). In addition, the partial correlation between Ka and mRNA expression, controlling for Ks, is small (r = -0.14, *P *= 7 × 10^-9^), whereas the partial correlation between Ks and expression, controlling for Ka, is significantly higher (r = -0.38, *P *< 10^-10^).

Although selection for optimal codons at the nucleotide level should significantly affect the rates of both synonymous and non-synonymous substitutions [40], there are additional constraints specifically acting on non-synonymous sites [42,43]. Many of these additional constraints affect the propensity of proteins to misfold and aggregate. For example, it has been reported that highly expressed *E. coli *proteins are more soluble than proteins with lower expression [44-46]. It is likely that the observed increase in solubility is necessary to avoid protein aggregation and non-functional binding [47] mediated by non-specific hydrophobic interactions. Using the genome-wide protein solubility data for *E. coli *proteins obtained by Niwa *et al*. [48], we indeed observed a significant correlation between solubility and expression (Figure 3c; Spearman's r = 0.27, *P *< 10^-10^). Importantly, the observed selection for solubility does not explain the correlation between the protein evolutionary rate and expression (Figure 3a; r = -0.45, *P *< 10^-10^); the partial correlation between Ka and expression, controlling either for solubility or for the fraction of charged residues, is still significant (r = -0.42 and -0.41, respectively; *P *< 10^-10^).

The positive correlation between solubility and expression is in agreement with an increase in the fraction of charged residues (Figure 3d; r = 0.28, *P *< 10^-10^) and a simultaneous decrease in the fraction of hydrophobic residues (r = -0.16, *P *< 10^-10^) in highly expressed *E. coli *proteins. We observed similar results by analyzing *E. coli *protein duplicates (paralogs) with different expression levels. By directly comparing duplicates expressed at different levels, many confounding factors, such as differences in folding topology or protein secondary structure, are removed. The analysis of 370 *E. coli *paralogs (see Materials and methods) demonstrated a decrease in the fraction of hydrophobic residues (paired Wilcoxon signed rank test, *P *= 7 × 10^-4^) and a simultaneous increase in the fraction of charged residues (*P *= 7 × 10^-6^) in the duplicates with higher expression levels.

**Figure 4:**
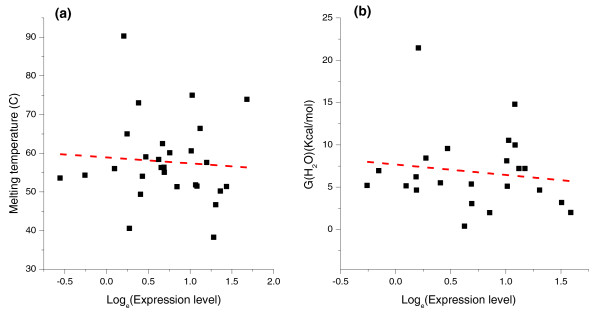
**Relationship between protein stability and mRNA expression**. The experimentally measured stability data were obtained from the ProTherm database [54], and the expression data for *E. coli *were obtained from the study by Lu *et al*. [78]. **(a) **Correlation between mRNA expression and melting temperature for 28 proteins (r = -0.14, *P *= 0.45). **(b) **Correlation between mRNA expression and folding free energy for 23 proteins (r = -0.08, *P *= 0.70). The dashed red line represents the linear regression between each variable and the natural logarithm of the expression values.

The analysis of 602 *E. coli *protein structures currently available in the PDB (see Materials and methods) confirmed a significant increase in the fraction of solvent-exposed charged residues in highly expressed proteins (r = 0.18, *P *= 6 × 10^-6^). While such an increase may lead to higher protein stabilities [49], a proposed consequence of selection for translational robustness [22], we did not detect strong correlations between mRNA expression and other structural features usually associated with increased protein stability [18,22]. For example, we did not observe a significant increase in the fraction of buried hydrophobic residues (r = 0.06, *P *= 0.13) [50-52] or an increase in the average number of contacts per residue (contact density) in highly expressed *E. coli *proteins (r = 0.02, *P *= 0.96). Neither did we find a decrease in the fraction of residues in loops or unstructured protein regions (r = 0.07, *P *= 0.06) [53]. Our analysis of experimentally determined *E. coli *protein stabilities assembled in the ProTherm database [54] also failed to reveal any significant correlation between protein stability, measured either by protein melting temperature (r = -0.14, *P *= 0.46) or folding free energy (ΔG, r = -0.08, *P *= 0.70), and mRNA expression level (Figure 4a,b). We also did not detect significant changes in the contact order, a structural measure strongly associated with folding speed [55,56], in highly expressed bacterial proteins (r = -0.01, *P *= 0.8).

Overall, the computational analysis described above suggests that, at least based on the currently available datasets, an increase in folding speed and/or protein stability for highly expressed bacterial proteins is unlikely to play a major role in constraining the protein molecular clock in *E. coli*.

## Discussion

The results presented here demonstrate that, at least in *E. coli*, the cost associated with the gratuitous expression of a protein is significantly higher than the additional toxicity cost incurred by destabilization or misfolding of the same amount of protein; by 'gratuitous' we imply here that the protein has no effect on fitness through its biological function. It is important to emphasize that our growth measurements are not sensitive enough to detect small fitness effects - for example, decreases in the growth rate on the order of 1% or less - and consequently we cannot rule out additional costs specifically related to misfolding toxicity [57]. In fact, a detailed study by Lindner *et al*. [58] using time-lapse microscopy showed that the presence of protein aggregates in *E. coli *has an effect on growth rate at the level of individual cells. Nevertheless, our experiments do show that the misfolding toxicity cost is significantly smaller than other costs associated with protein expression.

We believe that the main expression costs specifically in this bacterium are related to translational efficiency and jamming of the cell's cytoplasm with useless proteins. Importantly, expression costs associated with amino acid waste, or the energy required for gratuitous expression, were recently shown by Stoebel *et al*. [59] to play a relatively minor role. On the other hand, both gratuitous protein expression and suboptimal codons can significantly slow bacterial growth, for instance, by reducing the pool of free ribosomes in the cell [33,41]. This effect will preferentially affect highly expressed genes bound by a relatively larger number of ribosomes. A gene with non-optimal codons will slow the rate of translation (speed of ribosomal motion) and thus titrate more ribosomes. A reduced pool of free ribosomes will necessarily slow expression of all bacterial genes and thus decrease the rate of biomass synthesis [60].

Interestingly, we observed that bacteria expressing two of the mutants (F758S and G353D) grew significantly faster than cells expressing native LacZ protein (Figure 2a), although still not as fast as uninduced *E. coli*. This intriguing result demonstrates that titration of ribosomes cannot be the only explanation for the costs associated with gratuitous protein synthesis. The F758S and G353D proteins had the lowest catalytic activities of all constructs (Table 1) and both mutants, as well as I141N, were found mostly in inclusion bodies. It is likely that the localization of the LacZ proteins to inclusion bodies prevents jamming of the cytoplasm and relieves effects associated with non-functional binding. It was previously shown that an asymmetric partition of inclusion bodies during cell division may result in a cell rejuvenation phenotype [58]. We would like to emphasize that this result does not support the misfolding toxicity hypothesis, as these mutants grew faster than the strain expressing WT LacZ. Based on the growth rates of mutants primarily localized to inclusion bodies (V567D, F758S, I141N; average growth decrease 6.7%) and the proteins remaining in the cytoplasm (WT, V567D, A880E; average growth decrease 14%), one can conclude that effects of jamming and translational efficiency make approximately similar contributions to fitness.

An important separate question in the context of the mistranslation-induced misfolding hypothesis is whether phenotypic (transcriptional or translational) mutations can cause enough protein misfolding to be significantly cytotoxic. Although suboptimal codons are expected to substantially increase the translational error rate [39], no correlation was observed between codon optimization and the fraction of properly folded GFP by Kudla *et al. *[41]. Even if relatively rare, phenotypic mutations can still be significantly damaging if they occur in functionally and structurally important sties. This may explain a well-established correlation between codon optimization and evolutionary conservation of corresponding protein sites [61-63]. This correlation is not necessarily a consequence of selection against mistranslation-induced toxicity, and again may be primarily related to the loss of functional proteins and the cost of additional protein synthesis necessary to compensate for the misfolding. In fact, it has been reported that essential bacterial proteins have lower aggregation propensities than those predicted for non-essential proteins [46].

While our study demonstrates that misfolding toxicity is unlikely to be a universally dominant factor connecting expression and the protein molecular clock in all species, we cannot rule out the possibility that toxicity may play an important role in other species. We note, however, that in higher organisms the correlations between mRNA expression and the protein molecular clock are generally much weaker than in some microbes. For example, Liao *et al*. [64] demonstrated that expression plays a relatively minor role in constraining the molecular clock in mammalian species. Also, by comparing evolutionary rate of separate and fused protein domains in human and *Arabidopsis*, Wolf *et al*. [65] found a comparable contribution from expression and structural-functional constraints.

A number of elegant experimental studies have demonstrated a cytotoxic effect of several misfolded or marginally stable proteins in higher organisms [66,67]. For instance, several hundred mutations in the SOD1 protein were shown to result in aggregates associated with amyotrophic lateral sclerosis in humans [68]; also, non-natural peptides have been used to induce cytotoxic aggregates of GFP in *C. elegans *[69]. Although these studies directly demonstrate the importance of misfolding and aggregation for some specific proteins, the extent to which these effects dominate the molecular clock for all proteins in these and other species needs to be investigated and again compared to other contributing factors.

## Conclusions

Our experimental results suggest that selection against toxic protein misfolding is unlikely to be the universal and dominant factor determining the rate of the protein molecular clock in all species. We demonstrate that, at least in *E. coli*, other factors associated with gratuitous protein synthesis, such as translational efficiency and possibly jamming of the cytoplasm, are likely to be the primary constraints. Our computational analyses also suggest a relatively weaker, but statistically significant, selection for increasing solubility and polarity in highly expressed *E. coli *proteins.

## Materials and methods

### Strains and mutant generation

*E. coli *K12 strain GP4 (W3102, XA 21Z, *lac*I^q^) was used in all experiments. *lacZ *was expressed from the IPTG-inducible *lac *promoter in plasmid PIV18 [33]; PIV18 is a pBR322 derivative that carries a mutation in the Shine Dalgarno sequence of the *lacZ *transcript that increases translation efficiency. Site directed mutagenesis was carried out using Stratagene's QuikChange Lightning kit (Stratagene, Cedar Creek, TX, USA). pBR322 was used as the empty plasmid control.

### Growth curve analysis

For each construct, a sweep of colonies was grown overnight on Luria-Bertani (LB) liquid media supplemented with 100 μg/ml ampicillin. Overnight cultures were diluted by a 1:100 factor and grown on M9 minimal media suplemented with 0.5% casaminoacids, 0.25 μg/ml thiamine, 100 μg/ml ampicillin and either 0.4% glycerol or acetate as carbon sources. We transfered 300 μl of cells with an OD600 of 0.5 to flasks containing 5.5 ml of prewarmed media suplemented with the appropiate amount of IPTG. Two hours after induction, OD600 was measured every 45 minutes. Growth rate was determined as the regression line slope of time and the logarithm of OD600.

### SDS-PAGE and western blotting

The equivalent of 200 μl of cells at an OD600 of 0.7 was collected by centrifugation and lysed using Novagen's BugBuster (primary amine-free) Protein Extraction Reagent (Novagen, Merck, Darmstadt, Germany). Soluble proteins were retrieved after centrifugation of the lysed cells and aggregated proteins were then harvested following instructions for inclusion body purification described in the BugBuster reagent manual. Both fractions were saved in a 50 μl volume including 10 μl 4× SDS loading buffer, boiled, and electrophoresed on a 10% SDS polyacrylamide gel. Gels were stained with Coomassie blue and scanned for analysis. For the analysis of total protein, cells were lysed in BugBuster reagent containing rLysozyme and boiled after addition of 4× SDS loading buffer. Bands were quantified using the ImageJ program [70].

Protein samples separated by SDS-PAGE as described above were blotted overnight onto a nitrocellulose membrane and incubated with Anti-GroEL antibody produced in rabbit 1:10 000 (Sigma Aldrich, St Louis, MO, USA). Blots were blocked with 5% non-fat dry milk, incubated with 1:3,000 anti-rabbit horseradish peroxidase conjugate antibody and visualized with Amersham's ECL Plus Western Blotting Reagent (GE Healthcare, Munich, Germany).

### Structural analysis of *E. coli *proteins

In the analysis we used 602 *E. coli *protein structures currently available in the PDB [71]. To prevent sampling biases, we filtered available PDB entries so that no two protein structures used in the calculations had sequence identity higher than 90%; similar results were obtained without filtering. We defined buried residues as those with a solvent accessible area smaller than 16% [72,73]. Solvent accessibility was calculated by the DSSP [74] program. The fraction of protein residues in loops was also calculated using DSSP. Two non-adjacent protein residues were considered to be in contact if any two of their non-hydrogen atoms were closer than 4.5 Å [75]. The protein contact density was defined as the average number of non-adjacent contacts per residue. Contact order was calculated as (L × N)^-1 ^× ΣΔS_ij_, where N is the total number of contacts, L is the total number of residues in the protein and ΔS_ij_, which is summed over all contacts, is the number of amino acids separating contacting residues [56]. *In vitro *solubility data for *E. coli *proteins was obtained directly from the study of Niwa *et al*. [48].

### Correlation of the synonymous (Ks) and non-synonymous (Ka) substitution rates with expression

Orthologous open reading frames and protein sequences from *E. coli *and *Salmonella enterica *were used to calculate Ks and Ka values. The *E. coli-Salmonella *orthologs were determined as bi-directional best hits using protein BLAST [76]. Ka and Ks values were calculated using the maximum likelihood method implemented in the PAML package [77]. The mRNA expression data reported by Lu *et al*. [78] were used to calculate the correlations. For the analysis of duplicated genes, we defined duplicates as pairs of *E. coli *proteins having more than 40% sequence identity that could be aligned for at least 80% of their total length using BLAST. In the analysis of duplicates, we used expression data from 466 experiments in the Many Microbes Microarrays Database [79]. We selected for the analysis only the pairs for which one paralog had higher expression values in more than 80% of the reported experiments.

## Abbreviations

GF: green fluorescent protein; IPTG: isopropyl β-D-1-thiogalactopyranoside; Ka: the rate of non-synonymous substitutions; Ks: the rate of synonymous substitutions; PDB: Protein Data Bank; WT: wild type.

## Competing interests

The authors declare that they have no competing interests.

## Authors' contributions

GP carried out the experiments and computational analyses. DV, MG and GP conceived the experiments and analyzed the results. DV and GP wrote the paper with revisions by MG. All authors read and approved the final manuscript.
